# Hippocampal texture asymmetry features on ^18^F-FDG PET differentiating anti-LGI1 and anti-GABABR encephalitis

**DOI:** 10.3389/fimmu.2026.1878467

**Published:** 2026-08-25

**Authors:** Jian Pan, Xiaotong Li, Bo Zhuang, Tianren Zhang, Qianjiang Ding, Guifei Zhou, Guowei Yang, Xiaobin Zhao

**Affiliations:** 1 School of Information Engineering, Nantong Institute of Technology, Nantong, China; 2 Department of Nuclear Medicine, Beijing Tiantan Hospital, Capital Medical University, Beijing, China; 3 School of Information Science and Technology, Yunnan Normal University, Kunming, China; 4 Department of Radiology, The First Affiliated Hospital of Ningbo University, Ningbo, China

**Keywords:** anti-GABABR encephalitis, anti-LGI1 encephalitis, hippocampus, machine learning, radiomics

## Abstract

**Objective:**

This study aimed to investigate whether hippocampal texture asymmetry features extracted from PET could effectively differentiate anti-LGI1 encephalitis from anti-GABABR encephalitis based on a hippocampal laterality radiomics (HLR) model.

**Methods:**

A total of 82 patients (57 anti-LGI1, 25 anti-GABABR) were retrospectively enrolled. Radiomic texture features were extracted from the left, right and bilateral hippocampus. Asymmetry features were calculated based on hippocampal texture features, which were used to construct HLR model. In addition, a left hippocampal radiomics (LHR) model, a right hippocampal radiomics (RHR) model, and a bilateral hippocampal radiomics (BHR) model were constructed based on texture features of left, right and bilateral hippocampus, respectively. The leave-one-out cross-validation was utilized for hyperparameter tuning. Model performance was evaluated using receiver operating characteristic (ROC) curve analysis, decision curve analysis (DCA), integrated discrimination improvement (IDI), and net reclassification improvement (NRI), and SHAP feature analysis.

**Results:**

The HLR model achieved the best predictive performance, with an AUC of 0.898, accuracy of 84.21%, sensitivity of 81.82%, and specificity of 87.50%. The AUC of the HLR model was significantly higher than those of the LHR, RHR, and BHR models (*p* < 0.001, DeLong test). DCA demonstrated superior net clinical benefit for the HLR model across relevant thresholds, while IDI and NRI confirmed significant improvement over the BHR model (*p<* 0.01). Wavelet-filtered GLSZM and GLRLM asymmetry metrics were identified as primary predictors.

**Conclusions:**

Hippocampal texture asymmetry features may serve as feasible imaging biomarkers for differentiating anti-LGI1 encephalitis from anti-GABABR encephalitis.

## Introduction

Autoimmune encephalitis (AE) is an immune-mediated disease characterized by an immune-mediated damage of the central nervous system (1, 2). Anti-leucine-rich glioma-inactivated 1 (LGI1) antibody encephalitis and anti-gamma-aminobutyric acid B receptor (GABABR) antibody encephalitis are the two most common subtypes of autoimmune limbic encephalitis (3). The clinical presentations of this encephalitis are indicative of limbic system involvement, such as seizures, recent memory decline, and psychiatric or behavioral abnormalities. However, there are significant differences in clinical prognosis, related tumor risk (such as anti-GABABR encephalitis often accompanied by small cell lung cancer (4)), and treatment response. Therefore, early and accurate discrimination between anti-LGI1 and anti-GABABR encephalitis is crucial for facilitating individualized therapeutic strategies and improving clinical outcomes (5, 6).

For the anti-LGI1 and anti-GABABR encephalitis, the confirmatory diagnosis mainly depends on antibody testing. However, antibody testing is time-consuming and remains unavailable in some clinical settings (4), which may delay the optimal therapeutic window. Magnetic resonance imaging (MRI), a non-invasive and fast assessment tool, can initially diagnosis for preliminary treatment (4). Compared to MRI, the fluorine-18 fluorodeoxyglucose positron emission tomography (^18^F-FDG PET) is a sensitive functional modality for detecting anti-LGI1 and anti-GABABR encephalitis (7–9). In clinical practice, qualitative visual assessment or simple measurement of standardized uptake values is used to evaluate PET images to detect subtle metabolic abnormalities. However, the metabolic patterns are overlapped in these two subtypes, such as the hippocampus exhibits hypermetabolism, and the evaluation results are limited by the subjective experience of the observer (10, 11). Machine learning (ML) has increasingly been utilized to assess medical images and enhance diagnostics (12). Consequently, ML serves as a viable approach for differentiating between anti-LGI1 and anti-GABABR encephalitis utilizing PET imaging.

Radiomics, a technique extensively employed in medical imaging studies (13–16), can extract many quantitative features from medical images that are imperceptible to the naked eye, such as textural features. The extracted radiomic features can be utilized to construct predictive models that support precision diagnosis. Currently, radiomics has been widely applied in the study of brain diseases (17–19). However, many radiomics studies mainly extracted features from unilateral or bilateral hemispheres (20–22). As a result, the differential radiomic features between the two hemispheres, which essentially represent laterality-related characteristics, were often neglected. For autoimmune encephalitis, the associated metabolic and inflammatory changes frequently manifest as an interhemispheric imbalance, a phenomenon substantiated by asymmetric radiopharmaceutical uptake observed in PET imaging (23, 24). Asymmetry features derived from the differences in left-right hippocampal radiomics features can reveal disease-specific interhemispheric imbalances caused by encephalitis. Therefore, asymmetry features hold great potential for improving the performance of radiomics-based diagnostic models.

This study aimed to extract radiomic features from the left and right hippocampus and calculate the hippocampal texture asymmetry features to construct a hippocampal laterality radiomics (HLR) model to differentiate anti-LGI1 encephalitis from anti-GABABR encephalitis. We hypothesized that the hippocampal texture asymmetry features could capture differences of left and right hippocampal texture features, thereby offering clinicians a novel and efficient means for differential diagnosis.

## Materials and methods

### Overview of the radiomics framework

**Figure 1:**
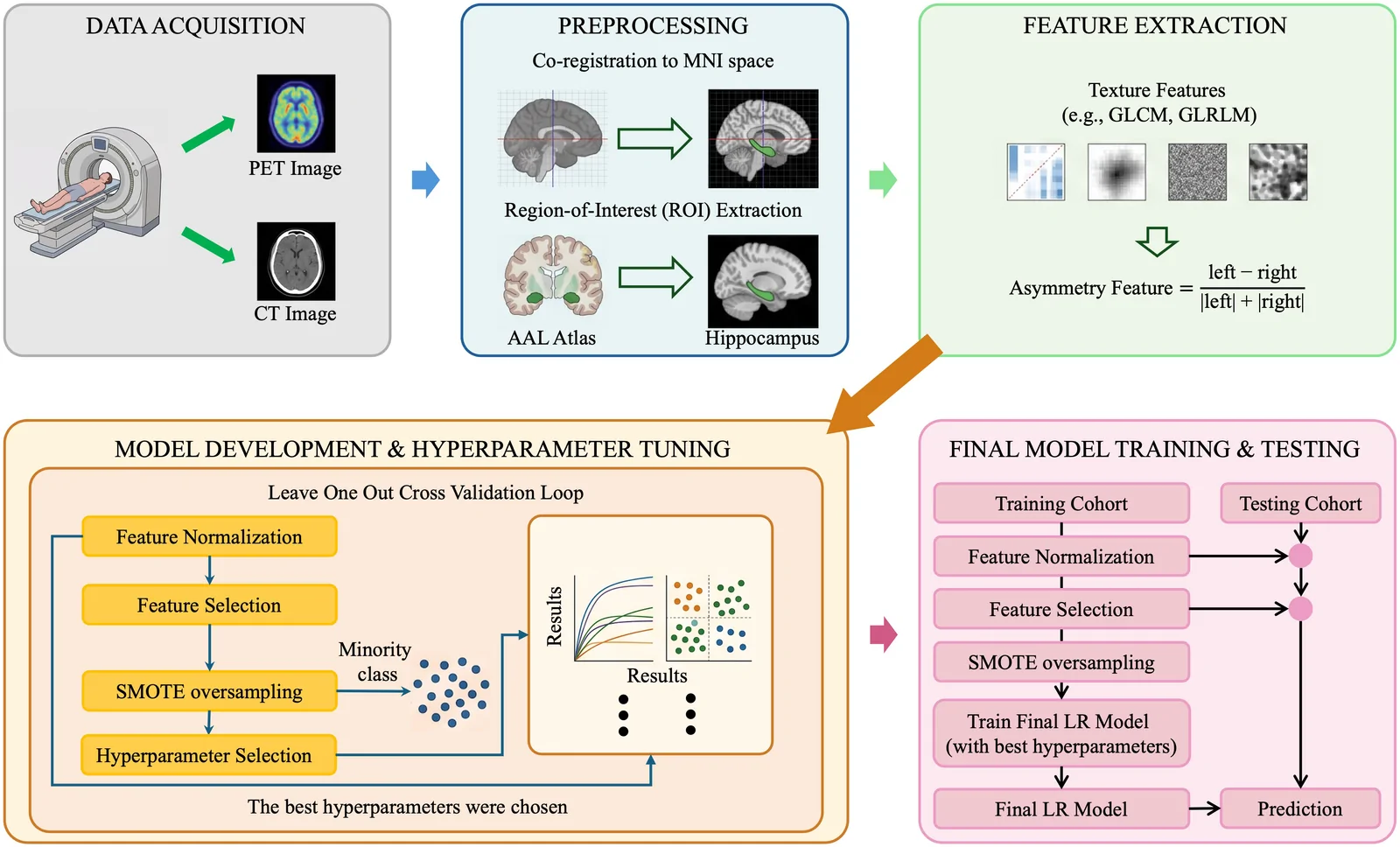
Schematic overview for differentiating anti-LGI1 and anti-GABABR encephalitis. The framework comprised five main components: Data Acquisition, Preprocessing, Feature Extraction, Model Development & Hyperparameter Tuning, and Final Model Training & Testing. AAL, Anatomical Automatic Labeling; CT, Computed Tomography; GLCM, Gray-Level Co-occurrence Matrix; GLRLM, Gray-Level Run Length Matrix; LOOCV, Leave-One-Out Cross-Validation; LR, Logistic Regression; MNI, Montreal Neurological Institute; PET, Positron Emission Tomography; ROI, Region of Interest; SMOTE, Synthetic Minority Over-sampling Technique.

As depicted in **Figure 1**, the overall procedure for differentiating anti-LGI1 encephalitis from anti-GABABR encephalitis based on PET/CT images consists of five main components: data acquisition, data preprocessing, feature extraction, model development and hyperparameter tuning, and final model training and testing.

### Participants

This study was approved by the Medical Ethics Committee of Beijing Tiantan Hospital and was conducted in accordance with the Declaration of Helsinki.

This study included a total of 82 patients with AE. To strictly evaluate the model developed in this study, patients were split into a training cohort and an independent testing cohort based on the collection date. Among them, 63 patients were collected in 2020 and assigned to the training cohort, while the remaining 19 patients were collected in 2026 and assigned to the testing cohort.

All patients included in this study had positive LGI1 or GABAB receptor antibodies in cerebrospinal fluid and/or serum, accompanied by clinical manifestations including memory impairment, seizures, and cognitive dysfunction. Furthermore, PET and CT images were necessary for examination. Patients with seizures resulting from brain structural lesions or other diseases were excluded.

### Image acquisition

All patients underwent brain ^18^F-FDG PET/CT scan to obtain PET and CT images. First of all, patients were required to fast for a minimum of 6 hours. Then, ^18^F-FDG (0.10–0.15 mCi/kg) was intravenously injected after normoglycemia was verified. Subsequently, patients were kept at rest in a darkened room for 30 minutes. Finally, brain PET/CT images were acquired using GE-Discovery 690 scanner. The PET images were reconstructed using the VPFXS method, which is a 3D ordered subset expectation maximization algorithm integrated with Time-of-Flight and Point Spread Function correction. The acquisition parameters for PET images were as follows: matrix = 192×192 pixel, voxel size = 1.5625×1.5625×3.2700 mm^3^, axial slices = 47.

### Image preprocessing

All images were preprocessed using SPM12 (Wellcome Trust Center for Neuroimaging, London, UK). First, CT images were co-registered to the corresponding PET images using SPM12. Second, the co-registered CT images were normalized to the Montreal Neurological Institute (MNI) template using the Clinical Toolbox, an extension of SPM12 (https://www.nitrc.org/projects/clinicaltbx/) (25). Third, the deformation field derived from CT normalization was applied to the PET images, thereby normalizing them to the MNI template. Then, the PET images were resampled to 2 × 2 × 2 mm^3^ using a 4th-degree B-spline interpolation. Fourth, the normalized PET images were smoothed using a 6-mm full-width-half-maximal isotropic Gaussian kernel. Fifth, the intensity of each voxel of each PET image was divided by the mean intensity calculated over the top 20% of voxels within that same PET image (26).

To rule out registration misalignments, the quality of spatial normalization was technically verified for all subjects through strict visual inspection across axial, coronal, and sagittal planes using SPM12’s ‘Check Reg’ interface, confirming precise anatomical alignment between temporal lobe boundaries and the MNI template. In addition, to avoid introducing high-frequency edge artifacts and altering spatial intensity correlations, which could compromise subsequent radiomic feature extraction, Partial-Volume Correction (PVC) was not applied in this study.

### Radiomic features extraction and asymmetry features calculation

To extract radiomics features from left, right and bilateral hippocampus, the corresponding masks were generated based on Anatomical Automatic Labeling (AAL) atlas (27–29). All radiomic features were extracted using pyradiomics (version 3.1.0) (30), an open-source library whose feature definitions, mathematical expressions, and extraction algorithms strictly comply with the Image Biomarker Standardization Initiative (IBSI) benchmarks. Prior to feature extraction, Square Root, Wavelet, Square, Gradient, Logarithm, 3D Local Binary Pattern, and Exponential were used as filters to process PET images. In addition, the range of discretization was calculated based on the pixel values of preprocessed PET image. Given that the intensity of PET image was normalized, the bin width was set to 0.1 in this study. Textural features of left, right and bilateral hippocampus were extracted from both the original and filtered images, yielding 1,275 features for each of the three regions per PET image.

To quantify the differences in texture features between the left and right hippocampus, the hippocampal texture asymmetry features were calculated based on the extracted radiomics features of the left and right hippocampus. Specifically, for each pair of similar texture features in the left and right hippocampus, the following formula is used to calculate the bias index:


$${\text{Feature}}_{\text{AF}}=\frac{{\text{Feature}}_{\text{left}}-{\text{Feature}}_{\text{right}}}{\left|{\text{Feature}}_{\text{left}}\right|+\left|{\text{Feature}}_{\text{right}}\right|}$$

where the Feature_left_ and Feature_right_ were the same texture feature value extracted from the left and right hippocampus, respectively. All paired texture features are calculated one by one using this method to obtain a set of biased features reflecting the asymmetry of bilateral hippocampal textures. To distinguish from the original radiomic features, the suffix “AF” (i.e., Asymmetry Feature) was appended to each original feature name.

### Classifier construction

The extracted hippocampal texture asymmetry features were used to construct the HLR model using logistic regression classifier. In addition, radiomics features of left, right and bilateral hippocampus were respectively used to construct left hippocampal radiomics (LHR) model, right hippocampal radiomics (RHR) model, and bilateral hippocampal radiomics (BHR) model, which were compared with the HLR model.

#### Hyperparameter optimization via leave-one-out cross-validation

Given that hyperparameter tuning for logistic regression classifier primarily focuses on the regularization parameter *C*, a total of 21 candidate *C* values (2^−10^ to 2^10^) were evaluated in this study. For each candidate *C*, this study performed leave-one-out cross-validation (LOOCV) and computed the corresponding accuracy as the selection criterion. The candidate *C* value that produced the maximum accuracy was selected as the optimal *C* value, and this value was subsequently used to construct the final classifier.

**Figure 2:**
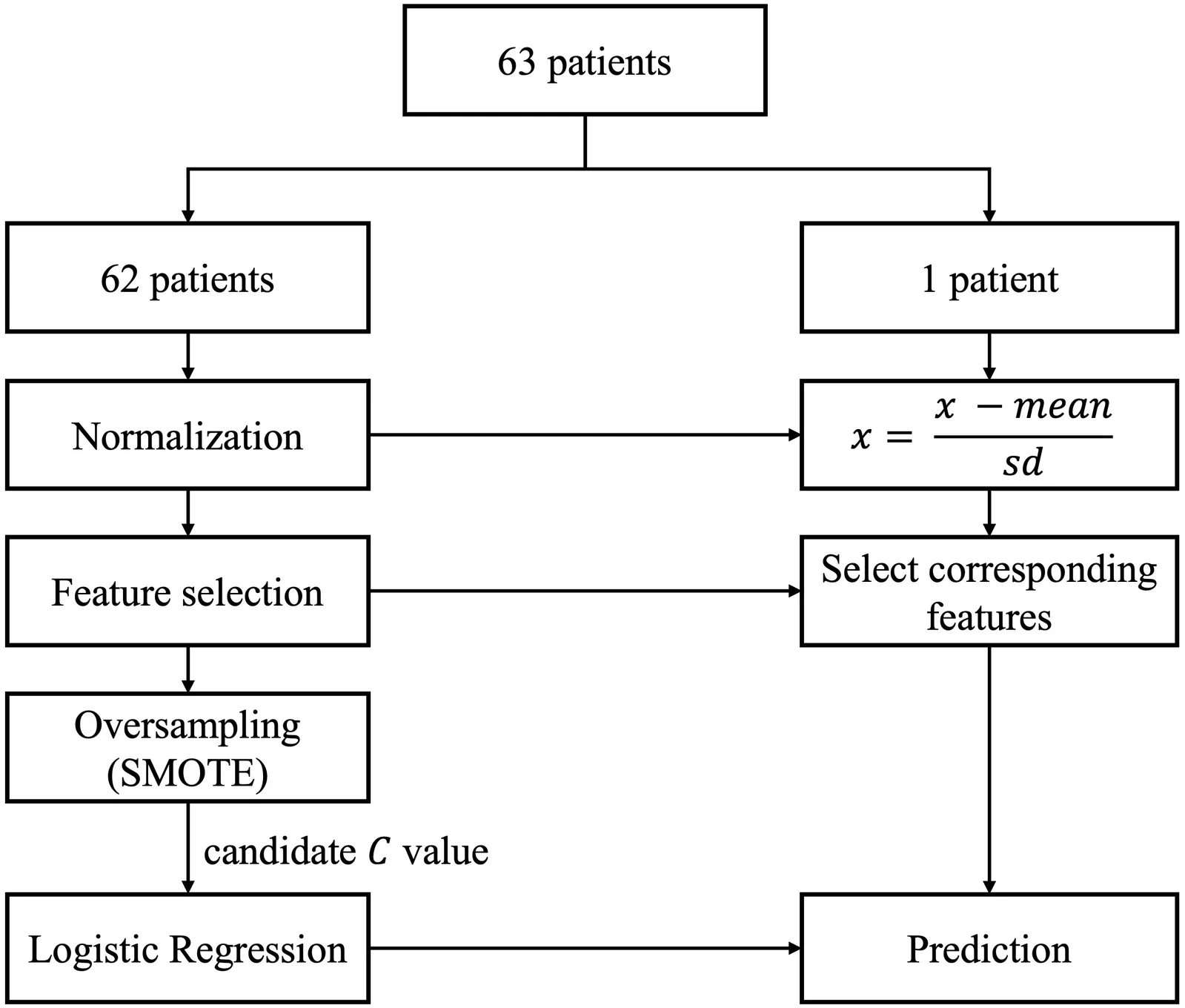
Detailed flowchart of the LOOCV scheme in the training cohort. The internal LOOCV pipeline was evaluated across the training cohort (n=63). In each iteration, 62 patients served as the training subset while 1 patient was withheld for validation. Z-score normalization and selected feature subsets derived from the 62-patient training subset were sequentially applied to the validation sample. SMOTE oversampling was implemented strictly within the training subset to address class imbalance prior to model fitting across candidate regularization hyperparameter *C* values. LOOCV, leave-one-out cross-validation; sd, standard deviation; SMOTE, Synthetic Minority Over-sampling Technique.

As shown in **Figure 2**, in each LOOCV loop, one sample was selected as validation set, while the remaining samples (i.e., n = 62) were utilized as training subset. To control the feature coefficients, the Z-score normalization was used to standardize feature values based on the mean and standard deviation of each feature in the training subset. The normalization parameters (mean and standard deviation) calculated from the training set were subsequently applied to the validation set.

To reduce the dimension of features, feature selection methods were conducted. The variance-based filtering was performed firstly. These features with zero variance were removed. Secondly, student’s *t*-test or Mann-Whitney U test was used to select features depending on data normality. Features with a *p* > 0.05 were excluded. Thirdly, to weaken the multicollinearity, the Pearson’s correlation analysis was used before features scaling. Based on Pearson’s correlation analysis, one of these features with absolute Pearson’s correlation coefficient greater than 0.9 were removed. Finally, the least absolute shrinkage and selection operator (LASSO) was employed for further feature selection.

To address the inherent class imbalance in the training data, the Synthetic Minority Over-sampling Technique (SMOTE) was employed in the training subset to overcome the problem of the imbalance between Anti-LGI1 and Anti-GABABR encephalitis. Then, a logistic regression classifier with the corresponding candidate *C* value was constructed using the processed training subset. The constructed classifier was used to predict the validation set.

After completing all iterations of LOOCV, all samples could obtain predictions which were used to calculate accuracy. This process was repeated for each of the 21 candidates, yielding 21 accuracy values. The candidate *C* that produced the highest accuracy was selected as the optimal hyperparameter and subsequently used to construct the final classifier.

#### Classifier construction and external validation

As shown in **Figure 1**, once the optimal *C* was selected through LOOCV, the final classifier was constructed and validated.

The final classifier was constructed using the entire training cohort. The raw asymmetry radiomic features were standardized using z-score normalization. The normalization parameters (mean and standard deviation) calculated from the entire training cohort were subsequently applied to the testing cohort. The feature selection pipeline was similar to that of LOOCV. The variance-based filtering, univariate analysis, correlation elimination, and LASSO were executed to determine the final feature subset (19 hippocampal texture asymmetry features). SMOTE was employed in the final feature subset to overcome the problem of the imbalance between Anti-LGI1 and Anti-GABABR encephalitis. The logistic regression classifier with the optimal *C* value was constructed using the final feature subset. The constructed logistic regression classifier was tested using the testing cohort.

To evaluate the potential impact of the sequence between feature selection and SMOTE on model performance, a sensitivity analysis comparing three pipelines, including Baseline (No SMOTE), SMOTE followed by feature selection (SMOTE-FS), and feature selection followed by SMOTE (FS-SMOTE), was conducted (**Supplementary Method 1**).

### Performance evaluation

The number of patients with anti-LGI1 encephalitis detected as anti-LGI1 encephalitis was referred to as true positive (TP). The number of patients with anti- GABABR encephalitis detected as anti-LGI1 encephalitis was referred to as false positive (FP). The number of patients with anti-GABABR encephalitis detected as anti-GABABR encephalitis was referred to as true negative (TN). The number of patients with anti-LGI1 encephalitis detected as anti- GABABR encephalitis was referred to as false negative (FN).

Therefore, accuracy was calculated by Equation 1

(1) $$\text{Accuracy}=\frac{\text{TN}+\text{TP}}{\text{TN}+\text{TP}+\text{FN}+\text{FP}}\times 100\%$$

sensitivity was calculated by Equation 2

(2) $$\text{Sensitivity}=\frac{\text{TP}}{\text{TP}+\text{FN}}\times 100\%$$

specificity was calculated by Equation 3

(3) $$\text{Specificity}=\frac{\text{TN}}{\text{TN}+\text{FP}}\times 100\%$$

The receiver operating characteristic (ROC) curves and precision-recall (PR) curves were plotted, and the area under the ROC (AUC) was computed to evaluate the performance of the classification models. DeLong test was utilized to assess statistical differences between four models in AUC values. Additionally, model goodness-of-fit was evaluated using calibration curves and Brier scores, and net clinical benefit was assessed across various threshold probabilities using decision curve analysis (DCA). Meanwhile, integrated discrimination improvement (IDI) and net reclassification improvement (NRI) were calculated to quantify the incremental predictive value of models. To provide robust non-parametric statistical inference, 95% confidence interval (CI) was estimated using non-parametric bootstrap.

### Feature importance analysis

To clarify the decision-making procedure of the HLR model and quantify the contributions of hippocampal texture asymmetry features to model predictions, Shapley Additive exPlanations (SHAP) was employed.

SHAP provides an additive feature attribution metric for each prediction by calculating the marginal contribution of each feature across all feasible feature combinations. For global interpretability, feature importance was ranked by computing the mean absolute SHAP value, thereby revealing the main factors influencing model performance. Furthermore, a SHAP summary plot was constructed to illustrate both the magnitude and directionality of feature impacts (i.e., whether high or low feature values positively or negatively influenced the predicted risk). Therefore, this dual strategy offered thorough local and global understanding of the behavior of the HLR model.

## Results

### Patient demographics

A total of 82 patients were enrolled in this study, comprising a training cohort (n=63) and a testing cohort (n=19). **Table 1** delineates the demographics of the patients of training cohort. In the training cohort, 46 patients were diagnosed with anti-LGI1 encephalitis (mean age 58.39 ± 11.84 years, 32 males) and 17 with anti-GABABR encephalitis (mean age 53.71 ± 10.47 years, 12 males). The age, gender, weight, and height of patients with anti-LGI1 encephalitis did not substantially differ from those of patients with anti-GABABR encephalitis, with *p* values of 0.16, 1.00, 0.81, and 0.73, respectively.

**Table 1:** Clinical characteristics of patients of training cohort.

Characteristic	Anti-LGI1 encephalitis	Anti-GABABR encephalitis	*p* value
Age(year), mean ± SD	58.39 ± 11.84 (n=46)	53.71 ± 10.47 (n=17)	0.16 ^a^
Gender (male/female)	32/14	12/5	1.00 ^b^
Weight (kg), mean ± SD	69.86 ± 11.63	70.65 ± 10.06	0.81 ^a^
Height (cm), mean ± SD	168.33 ± 7.64	169.06 ± 7.36	0.73^a^

LGI1, leucine-rich glioma-inactivated 1; GABAB, gamma-aminobutyric-acid B; SD, standard deviation.

a Independent samples t-test.

b Fisher’s exact test.

**Table 2** delineates the demographics of the patients of testing cohort. The testing cohort included 11 patients with anti-LGI1 encephalitis (mean age 59.27 ± 15.42 years, 9 males) and 8 patients with anti-GABABR encephalitis (mean age 53.13 ± 13.22 years, 5 males). The age, gender, weight, and height of patients with anti-LGI1 encephalitis did not substantially differ from those of patients with anti-GABABR encephalitis, with *p* values of 0.37, 0.60, 0.29, and 0.59, respectively.

**Table 2:** Clinical characteristics of patients of testing cohort.

Characteristic	Anti-LGI1 encephalitis	Anti-GABABR encephalitis	*p* value
Age(year), mean ± SD	59.27 ± 15.42 (n=11)	53.13 ± 13.22 (n=8)	0.37 ^a^
Gender (male/female)	9/2	5/3	0.60 ^b^
Weight (kg), mean ± SD	73.55 ± 10.59	67.63 ± 12.82	0.29 ^a^
Height (cm), mean ± SD	170.36 ± 4.34	168.38 ± 9.38	0.59^a^

LGI1, leucine-rich glioma-inactivated 1; GABAB, gamma-aminobutyric-acid B; SD, standard deviation.

a Independent samples t-test.

b Fisher’s exact test.

### Model performance

**Figure 3:**
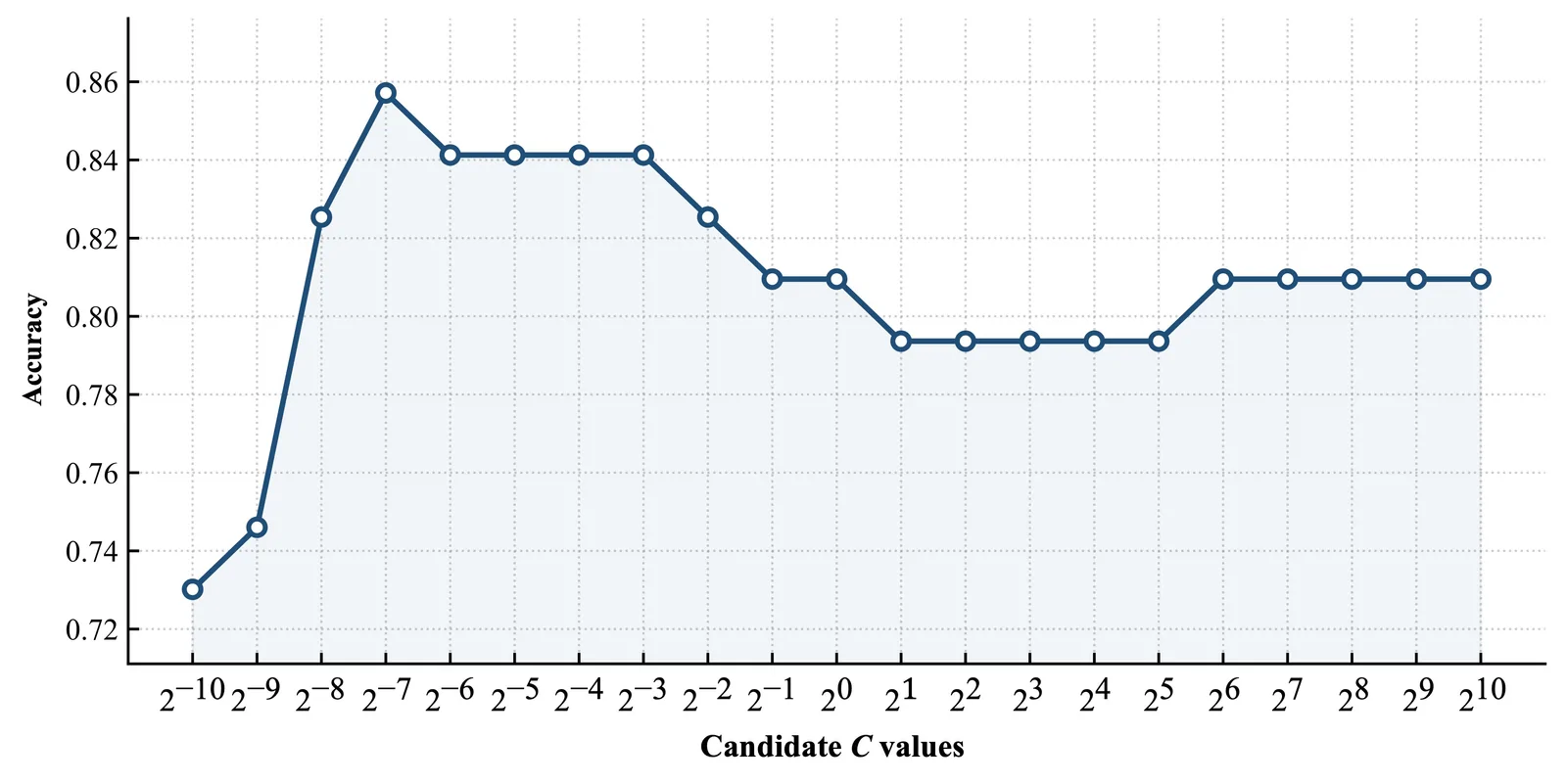
Hyperparameter optimization curve for the logistic regression classifier using LOOCV. The model accuracy was evaluated across 21 candidate regularization hyperparameter *C* values (ranging from 2^−10^ to 2^10^) during LOOCV. LOOCV, leave-one-out cross-validation.

As illustrated in **Figure 3**, hyperparameter tuning by LOOCV revealed that the classifier achieved its optimal performance with an accuracy of 85.71% at *C* = 2^−7^. Thus, this value was selected to construct the final classifier.

Based on optimal *C*, the HLR model was constructed using entire training cohort. The performances of the HLR model were presented in **Table 3**. Compared to LHR, RHR, BHR models, the HLR model achieved the highest performance, yielding an AUC of 0.898 (95% CI: 0.743-1.000). According to the DeLong test, the AUC of the HLR model was significantly superior to those of the LHR, RHR, and BHR models (all *p<* 0.001). In addition, the HLR model also demonstrated superior overall performance, with an accuracy of 84.21% (0.632-1.000), a sensitivity of 81.82% (0.556-1.000), a specificity of 87.50% (0.571-1.000), and the lowest Brier score of 0.157 (0.103-0.217). As shown in **Supplementary Table 1**, FS-SMOTE model (i.e., our HLR model) outperformed both the baseline model without SMOTE (AUC 0.841, accuracy 73.68%) and the SMOTE-FS model (AUC 0.830, accuracy 78.95%).

**Table 3:** Diagnostic performance of the hippocampal radiomics models.

Model name	AUC (95% CI)	Delong	Accuracy (%)	Sensitivity (%)	Specificity (%)	Brier score
LHR model	0.716 (0.472-0.960)	<0.001	57.89% (0.316-0.790)	63.64% (0.333-0.909)	50.00% (0.143-0.875)	0.216 (0.143-0.295)
RHR model	0.682 (0.425-0.939)	<0.001	63.16% (0.421-0.842)	54.55% (0.250-0.846)	75.00% (0.400-1.000)	0.298 (0.132-0.467)
BHR model	0.705 (0.443-0.966)	<0.001	68.42% (0.474-0.895)	63.64% (0.364-0.900)	75.00% (0.400-1.000)	0.234 (0.203-0.264)
HLR model	0.898 (0.743-1.000)	reference	84.21% (0.632-1.000)	81.82% (0.556-1.000)	87.50% (0.571-1.000)	0.157 (0.103-0.217)

LHR model: Left hippocampal radiomics model. RHR model: Right hippocampal radiomics model, BHR model: Bilateral hippocampal radiomics model, HLR model: Hippocampal laterality radiomics model.

In contrast, the single-sided hippocampal radiomics models exhibited lower diagnostic accuracy. Specifically, the LHR model achieved an AUC of 0.716 (95% CI: 0.472-0.960), an accuracy of 57.89% (0.316-0.790), a sensitivity of 63.64% (0.333-0.909), a specificity of 50.00% (0.143-0.875), and a Brier score of 0.216 (0.143-0.295). The RHR model yielded an AUC of 0.682 (95% CI: 0.425-0.939), an accuracy of 63.16% (0.421-0.842), a sensitivity of 54.55% (0.250-0.846), a specificity of 75.00% (0.400-1.000), and a Brier score of 0.298 (0.132-0.467). Compared with LHR and RHR models, the BHR model achieved better performance, with an AUC of 0.705 (95% CI: 0.443-0.966), an accuracy of 68.42% (0.474-0.895), a sensitivity of 63.64% (0.364-0.900), a specificity of 75.00% (0.400-1.000), and a Brier score of 0.234 (0.203-0.264).

**Figure 4:**
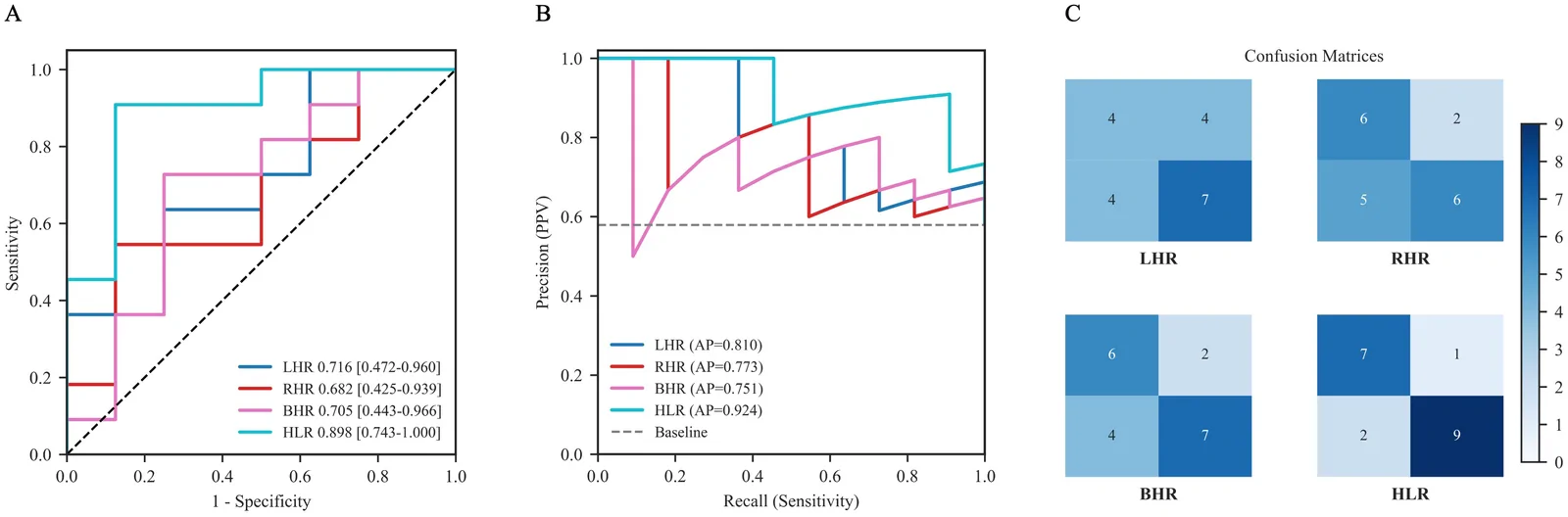
Diagnostic performance evaluation of the four models in the testing cohort. **(A)** ROC curves with corresponding AUC values and 95% CI. **(B)** PR curves presenting AP metrics relative to the random baseline. **(C)** Confusion matrices showing actual versus predicted patient classifications for LHR, RHR, BHR, and HLR models. AP, Average Precision; AUC, Area Under the Curve; BHR, Bilateral Hippocampal Radiomics; HLR, Hippocampal Laterality Radiomics; LHR, Left Hippocampal Radiomics; RHR, Right Hippocampal Radiomics; CI, confidence interval.

The ROC curves, PR curves and confusion matrices of the four models in the testing cohort are illustrated in **Figure 4**. As depicted by the ROC curves (**Figure 4A**), the HLR model achieved superior performance over the other three models, yielding the highest AUC. The PR curves further confirmed the robust performance of the HLR model (**Figure 4B**). The HLR model achieved the highest average precision (AP) of 0.924, outperforming the LHR (AP = 0.810), RHR (AP = 0.773), and BHR (AP = 0.751) models, all of which remained consistently above the random baseline.

Furthermore, the confusion matrices for the testing cohort (n=19) detailed the specific classification outcomes of each model (**Figure 4C**). The HLR model achieved the highest accuracy and only 3 misclassifications. In contrast, the LHR, RHR, and BHR models yielded 8, 7, and 6 misclassifications, respectively.

### Decision curve analysis of model performance

**Figure 5:**
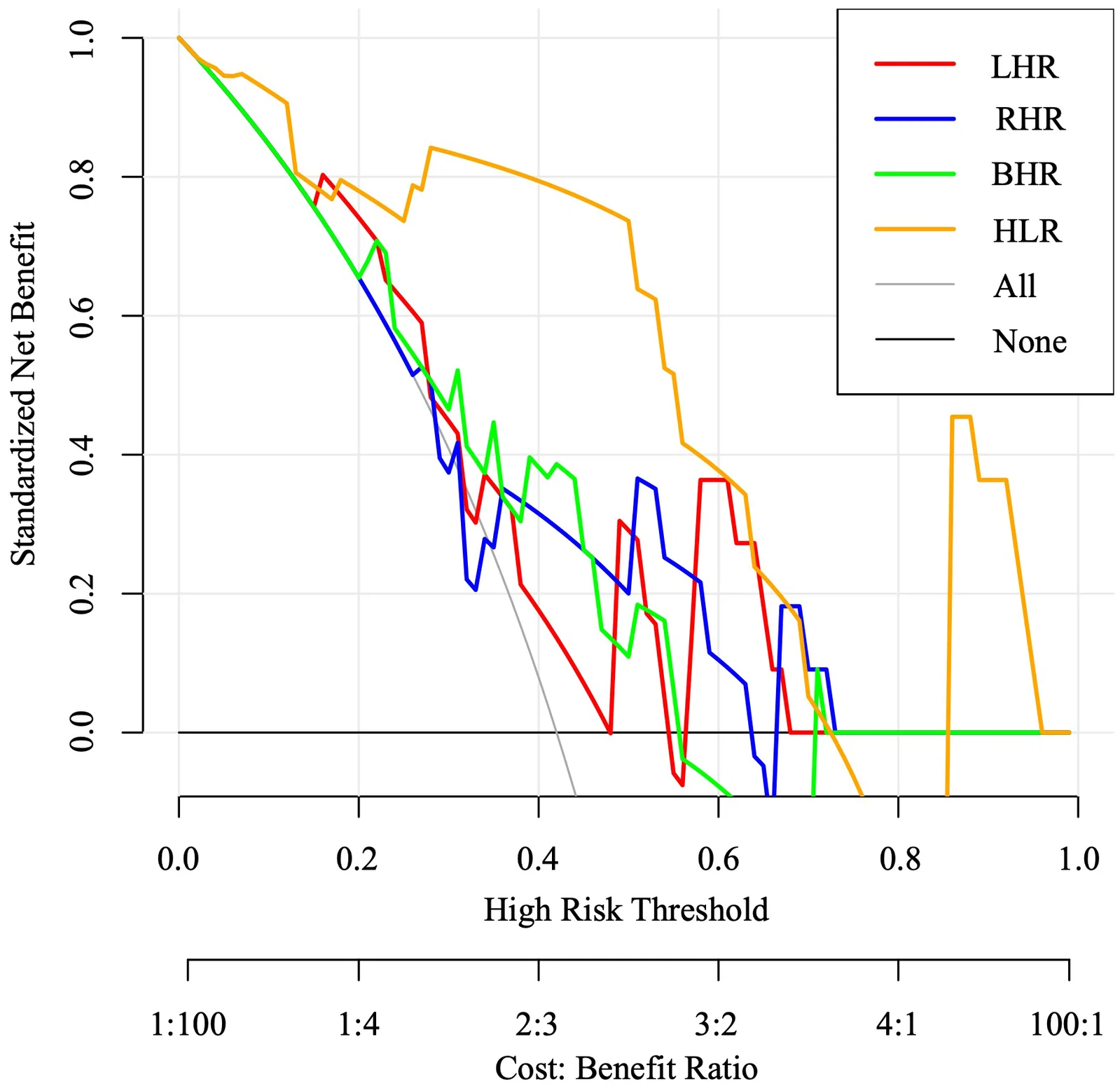
Decision curve analysis of the four models. The DCA was evaluated standardized net clinical benefit across varying high-risk threshold probabilities (and corresponding cost-benefit ratios). The HLR model (orange line) demonstrated superior standardized net benefit across a broad range of clinically relevant risk thresholds compared with the LHR (red), RHR (blue), BHR (green) models, as well as the default “treat-all” (gray line) and “treat-none” (black horizontal line) strategies. BHR, Bilateral Hippocampal Radiomics; HLR, Hippocampal Laterality Radiomics; LHR, Left Hippocampal Radiomics; RHR, Right Hippocampal Radiomics.

To evaluate the clinical utility of the developed HLR model, the DCA was performed. As shown in **Figure 5**, the DCA demonstrated that the HLR model consistently achieved the highest overall standardized net benefit across a broad range of high-risk threshold probabilities. Notably, within the threshold ranges of approximately 0.02 - 0.75 and 0.86 - 0.96, the HLR curve remained predominantly above those of the LHR, RHR, and BHR models, as well as the extreme “treat-all” and “treat-none” strategies, indicating that guiding clinical decisions with the HLR model yields greater potential net clinical gain.

### Model calibration and internal validation

**Figure 6:**
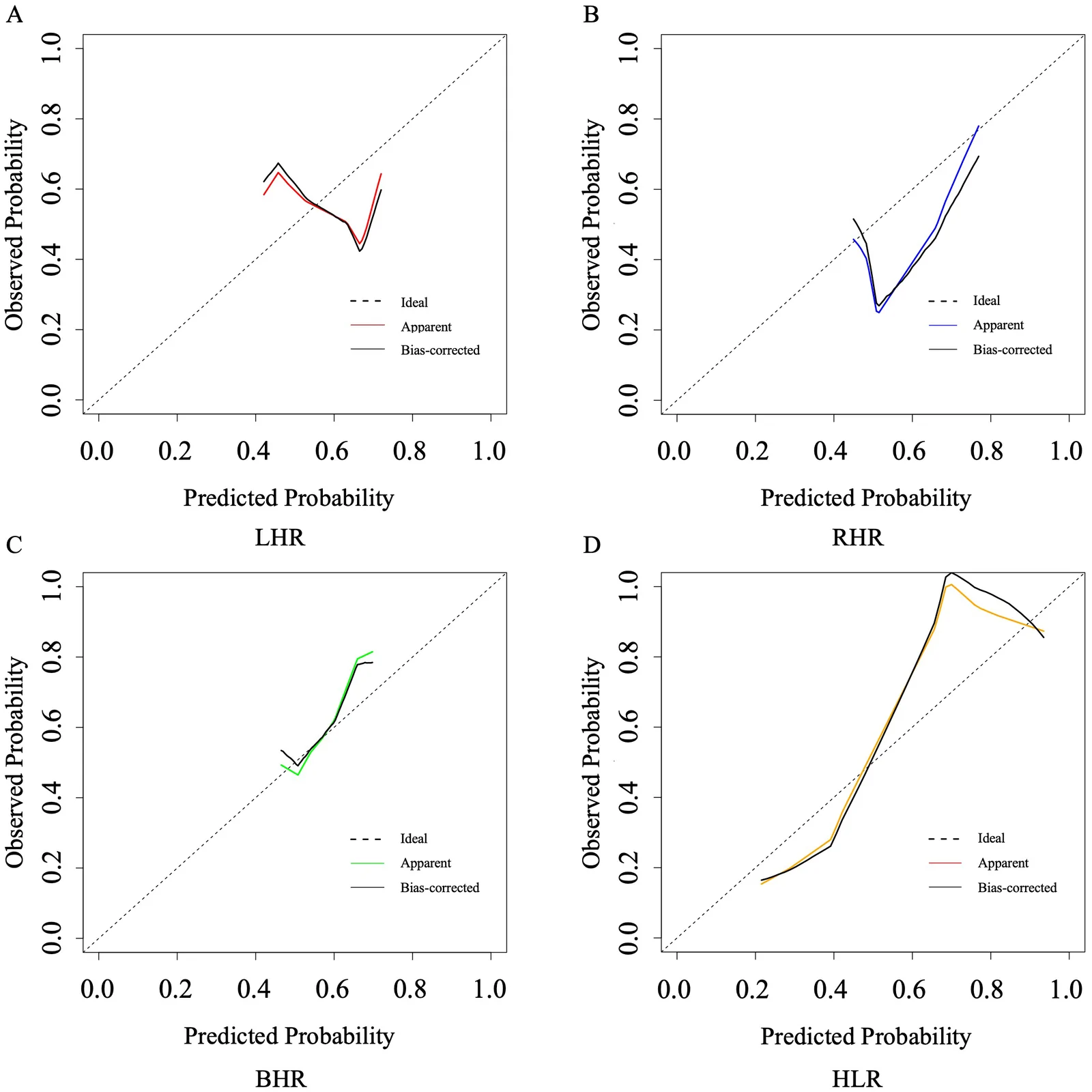
Calibration curves of the four models in the testing cohort. Calibration plots showed predicted probabilities versus actual observed outcomes for **(A)** LHR, **(B)** RHR, **(C)** BHR, and **(D)** HLR models. The dashed line represented ideal calibration (y = x). Colored solid lines represented apparent performance, and black solid lines represented bias-corrected calibration. BHR, Bilateral Hippocampal Radiomics; HLR, Hippocampal Laterality Radiomics; LHR, Left Hippocampal Radiomics; RHR, Right Hippocampal Radiomics.

The calibration performance of the four models was evaluated using calibration curves (**Figure 6**). Among the evaluated models, the HLR model (**Figure 6D**) demonstrated the most favorable overall calibration performance and the broadest dynamic range of predicted probabilities (approximately 0.20 to 0.95). The apparent curve and the bias-corrected curve for the HLR model showed near-perfect overlap across the prediction spectrum, indicating robust internal stability and minimal overfitting. The predicted probabilities of the HLR model aligned well with actual observed outcomes, despite slight miscalibration (underestimation of risk) in the higher predicted probability interval (0.70 to 0.90).

In contrast, the remaining three models exhibited suboptimal calibration characteristics (**Figure 6A**-6C). The LHR (**Figure 6A**), RHR (**Figure 6B**), and BHR (**Figure 6C**) models were constrained by restricted prediction risk ranges (predominantly clustered between 0.40 and 0.75). Additionally, these models displayed pronounced local fluctuations and substantial departures from the ideal reference line, such as a sharp V-shaped dip around a predicted probability of 0.50 in the RHR model.

### Incremental predictive value of the HLR model

**Figure 7:**
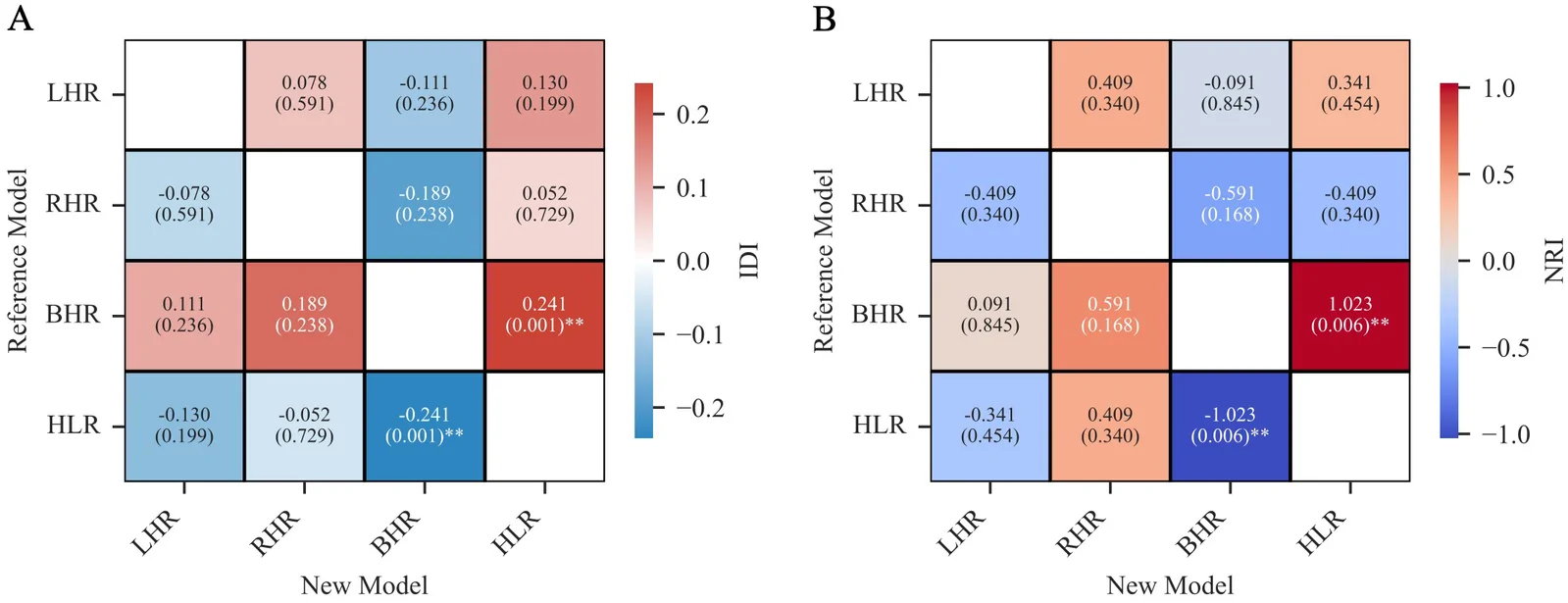
Pairwise comparisons of discrimination and reclassification metrics across models. Heatmaps illustrated pairwise model performance comparisons using **(A)** IDI and **(B)** NRI. Matrix rows represented reference models and columns represent comparison (new) models. BHR, Bilateral Hippocampal Radiomics; HLR, Hippocampal Laterality Radiomics; LHR, Left Hippocampal Radiomics; RHR, Right Hippocampal Radiomics; IDI, Integrated Discrimination Improvement; NRI, Net Reclassification Improvement.

To further quantify the incremental predictive value of incorporating hippocampal laterality radiomics features, pairwise comparisons of the NRI and IDI were performed among all four models (**Figure 7**).

When using the BHR model as the reference, the HLR model yielded a statistically significant enhancement in model performance. Specifically, the HLR model demonstrated a substantial discrimination improvement with an IDI of 0.241 (95% CI: 0.095 - 0.388, *p* = 0.001) and a significant net reclassification improvement with an NRI of 1.023 (95% CI: 0.292 - 1.753, *p* = 0.006) over the BHR model.

Although the NRI and IDI did not reach statistical significance when comparing the HLR model with the reference LHR model, both metrics yielded positive values. This suggests a positive trend toward improved reclassification and discrimination performance in the HLR model relative to LHR. Conversely, when using the RHR model as the reference, the HLR model exhibited a negative NRI, indicating a negative reclassification improvement.

### Feature importance and model interpretability using SHAP analysis

**Figure 8:**
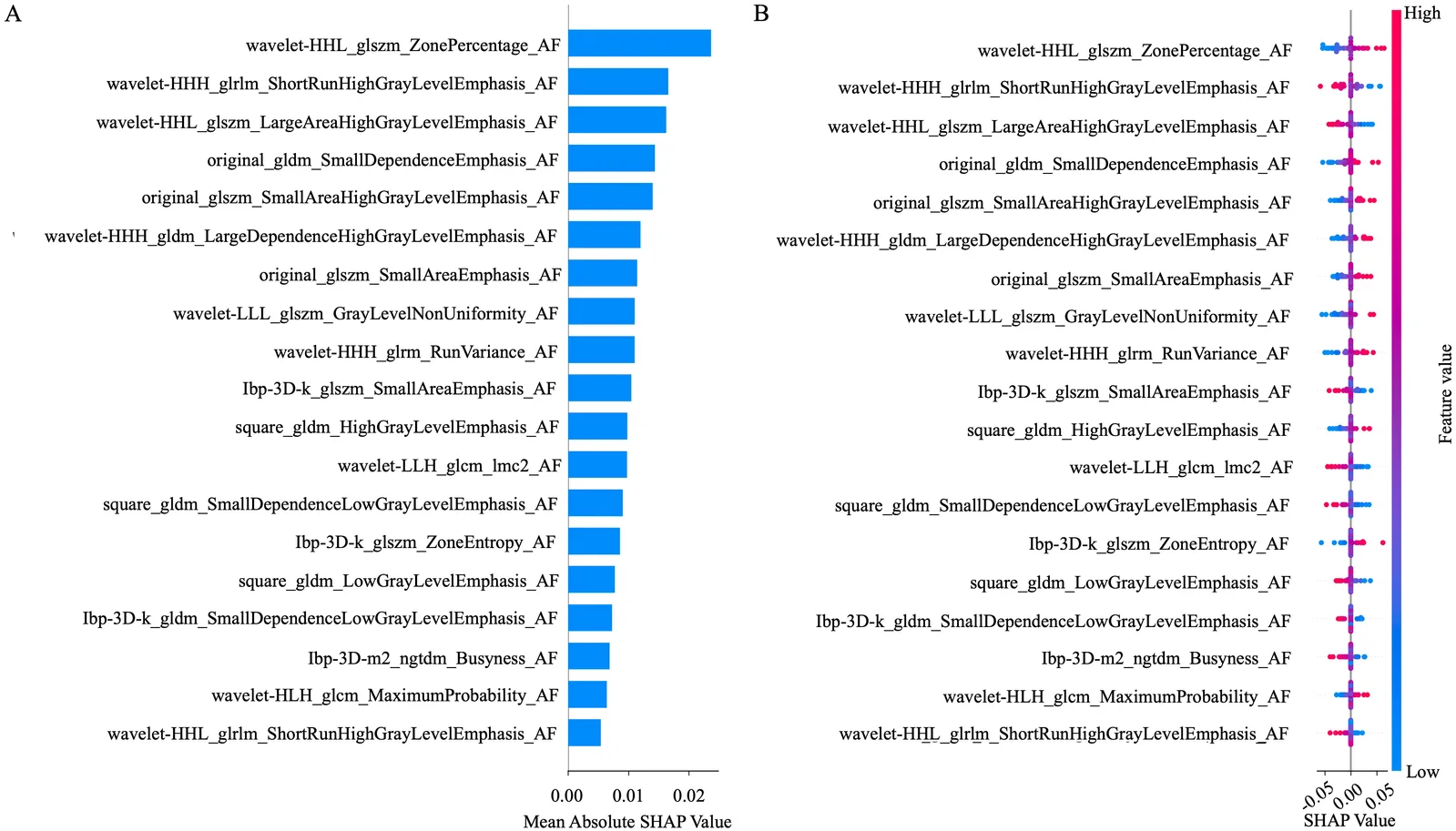
Feature importance and model interpretability of the HLR model using SHAP analysis. **(A)** Global feature importance ranking showing the top texture asymmetry features sorted by mean absolute SHAP value. **(B)** SHAP summary plot illustrating the directional impact of feature values on model predictions. Each dot represents a single patient, with dot colors indicating relative feature values (blue for low, red for high) and the horizontal axis showing the corresponding SHAP value. SHAP, Shapley Additive exPlanations.

A subset of 19 hippocampal texture asymmetry features was selected. To enhance model transparency and elucidate the contribution of individual asymmetry features to the final prediction, Shapley Additive exPlanations (SHAP) analysis was conducted (**Figure 8**).

The global feature importance ranking (**Figure 8A**) demonstrates the top features sorted by their mean absolute SHAP values. Among all evaluated asymmetry features, wavelet-HHL_glszm_ZonePercentage_AF was identified as the most influential predictor, exhibiting the highest mean absolute SHAP value. This was closely followed by wavelet-HHH_glrlm_ShortRunHighGrayLevelEmphasis_AF, and wavelet-HHL_glszm_LargeAreaHighGrayLevelEmphasis_AF.

The SHAP summary plot (**Figure 8B**) further illustrates the directional relationship between feature values and model outputs. For the leading feature, wavelet-HHL_glszm_ZonePercentage_AF, higher feature values (indicated by red dots) consistently corresponded to positive SHAP values, demonstrating that an increase in this feature value positively drove the model toward predicting the positive target outcome. Similarly, higher values of wavelet-HHH_glrlm_ShortRunHighGrayLevelEmphasis_AF and wavelet-HHL_glszm_LargeAreaHighGrayLevelEmphasis_AF predominantly exerted important impacts on the model’s prediction, affirming their robust discriminatory contributions.

## Discussion

In this study, we aimed to investigate whether hippocampal texture asymmetry features extracted from ^18^F-FDG PET could effectively differentiate anti-LGI1 encephalitis from anti-GABABR encephalitis based on a HLR model. Compared with models constructed using unilateral or bilateral hippocampal texture features, the HLR model achieved significantly better classification performance. Specifically, the HLR model achieved an AUC of 0.898, with accuracy, sensitivity, and specificity of 84.21%, 81.82%, and 87.50%, respectively. These results indicated that quantitative interhemispheric texture asymmetry provided significant incremental diagnostic value for distinguishing between these two autoimmune encephalitis subtypes.

Asymmetry features have been preliminarily applied in brain disease diagnosis such as neurodegenerative diseases and epilepsy (31–34). However, few studies were applied asymmetry features in distinguish between anti-LGI1 encephalitis and anti-GABABR encephalitis. The superior performance of the HLR model in the current study is mainly attributable to hippocampal texture asymmetry features, which directly quantify the relative textural deviation between the left and right hippocampus. Compared with the HLR model, the LHR and RHR models, which relied solely on unilateral radiomic information, achieved inferior classification performance because these models ignored potential disease-specific contralateral alterations. Therefore, hippocampal texture asymmetry features, by capturing the textural differences between the left and right hippocampus, have the potential to become imaging biomarkers for distinguishing anti-LGI1 encephalitis and anti-GABABR encephalitis. Previous studies have also demonstrated that asymmetric FDG uptake in anti-LGI1 encephalitis (23, 24) and anti-GABABR encephalitis (35) is frequently located in the hippocampus, which provided a biological rationale for the feature construction strategy of this study.

Compared with the LHR model, the RHR model achieved higher overall accuracy. Notably, these two unilateral models displayed complementary diagnostic characteristics: the LHR model yielded higher sensitivity, indicating superior capability of left hippocampal features in identifying anti-LGI1 encephalitis, whereas the RHR model demonstrated higher specificity, suggesting that right hippocampal features are more advantageous for recognizing anti-GABABR encephalitis. Although the BHR model outperformed both unilateral models by incorporating features from both sides, its performance gain remained marginal. This modest improvement likely stemmed from the fact that directly aggregating bilateral features without explicit side-to-side comparison failed to capture interhemispheric gradients and might have introduced non-informative spatial noise. These findings indirectly suggested that asymmetric features that quantified the relationship between left and right hippocampal features, which could improve the performance of diagnosis of anti-LGI1 encephalitis and anti-GABABR encephalitis.

Multi-dimensional evaluation across DCA, NRI, and IDI further confirmed the clinical utility of the HLR model. Beyond quantitative performance metrics, accurate distinction between anti-LGI1 and anti-GABABR encephalitis holds vital clinical implications for therapeutic decision-making and disease management. Anti-GABABR encephalitis is strongly associated with underlying paraneoplastic etiologies, particularly small cell lung cancer, which is present in up to 50% of cases (4). Misclassifying anti-GABABR encephalitis as anti-LGI1 encephalitis could lead to the omission or delayed initiation of rigorous systemic tumor surveillance (e.g., chest CT or bronchoscopy), potentially allowing occult malignancies to progress undetected during the critical therapeutic window. Conversely, misclassifying anti-LGI1 encephalitis as anti-GABABR encephalitis might trigger unnecessary, costly, and invasive oncological workups, causing substantial patient anxiety and potentially delaying prompt immunotherapy, which is essential for rapidly suppressing faciobrachial dystonic seizures and preventing irreversible cognitive impairment or hippocampal sclerosis (36, 37). Therefore, the high specificity (87.50%) and sensitivity (81.82%) of the HLR model offer meaningful decision support in optimizing oncological screening protocols and individualized immunotherapy strategies.

In our SHAP feature importance analysis, several wavelet-filtered hippocampal texture asymmetry features emerged as key drivers of the HLR model, particularly metrics derived from the GLSZM and GLRLM. When calculated as asymmetry features, these features explicitly capture interhemispheric dissimilarity in spatial cluster size, run lengths, and intensity distributions between the left and right hippocampus. Variations in these asymmetry values describe a side-to-side divergence in spatial metabolic texture patterns. It is crucial to emphasize that these radiomic features are fundamentally mathematical and statistical representations of spatial voxel intensity distributions derived from PET image matrices. Although spatial variations in FDG uptake are known to occur in autoimmune encephalitis, these radiomic metrics should be viewed as macroscopic imaging phenotypes rather than direct representations of specific immunopathological mechanisms, cellular changes, or tissue microenvironments. Consequently, these textural asymmetry metrics serve solely as exploratory imaging markers, and further multi-modal imaging, biological, or histopathological correlations are necessary to clarify their potential clinical and biological underpinnings.

Several limitations should be recognized. First, the sample size was comparatively modest. The prevalence and incidence of anti-LGI1 encephalitis and anti-GABABR encephalitis are relatively low (38, 39). Second, there was an imbalance in the number of patients between anti-LGI1 encephalitis and anti-GABABR encephalitis. External validation in larger, independent cohorts is warranted. Third, due to incomplete clinical data in a subset of patients, a standalone clinical model could not be established for direct comparison with the HLR model. Future studies with prospectively collected, complete clinical and imaging data will be conducted to validate our findings. Fourth, to avoid introducing high-frequency edge artifacts and altering spatial intensity correlations, PVC was not applied in this study. However, partial-volume effects may affect the radiomic features of the hippocampus. Future studies will explore the impact of the PVC algorithms on the radiomic features of the hippocampus. Finally, AAL atlas-based hippocampal ROIs might fail to address fine individual anatomical variations. Future studies will leverage deep learning algorithms to achieve automated and patient-specific hippocampal segmentation.

In conclusion, this study proposed a hippocampal texture asymmetry features-driven HLR model, which demonstrated strong diagnostic performance in distinguishing between anti-LGI1 encephalitis and anti-GABABR encephalitis. By capturing interhemispheric metabolic heterogeneity, the HLR model offers a promising quantitative tool to support clinical decision-making. Future prospective studies with larger, multi-center cohorts are needed to further validate its clinical utility and generalizability.

## Data Availability

The original contributions presented in the study are included in the article/**Supplementary Material**. Further inquiries can be directed to the corresponding authors.

## References

[1] Lancaster E Dalmau J . Neuronal autoantigens--pathogenesis, associated disorders and antibody testing. Nat Rev Neurol. (2012) 8:380–90. doi: 10.1038/nrneurol.2012.99 22710628 PMC3718498

[2] Seery N Butzkueven H O'Brien TJ Monif M . Rare antibody-mediated and seronegative autoimmune encephalitis: An update. Autoimmun Rev. (2022) 21:103118. doi: 10.1016/j.autrev.2022.103118 35595048

[3] Heine J Pruss H Bartsch T Ploner CJ Paul F Finke C . Imaging of autoimmune encephalitis--Relevance for clinical practice and hippocampal function. Neuroscience. (2015) 309:68–83. doi: 10.1016/j.neuroscience.2015.05.037 26012492

[4] Graus F Titulaer MJ Balu R Benseler S Bien CG Cellucci T . A clinical approach to diagnosis of autoimmune encephalitis. Lancet Neurol. (2016) 15:391–404. doi: 10.1016/s1474-4422(15)00401-9 26906964 PMC5066574

[5] Ances BM Vitaliani R Taylor RA Liebeskind DS Voloschin A Houghton DJ . Treatment-responsive limbic encephalitis identified by neuropil antibodies: MRI and PET correlates. Brain. (2005) 128:1764–77. doi: 10.1093/brain/awh526 15888538 PMC1939694

[6] Vincent A Buckley C Schott JM Baker I Dewar BK Detert N . Potassium channel antibody-associated encephalopathy: a potentially immunotherapy-responsive form of limbic encephalitis. Brain. (2004) 127:701–12. doi: 10.1093/brain/awh077 14960497

[7] Solnes LB Jones KM Rowe SP Pattanayak P Nalluri A Venkatesan A . Diagnostic value of (18)F-FDG PET/CT versus MRI in the setting of antibody-specific autoimmune encephalitis. J Nucl Med. (2017) 58:1307–13. doi: 10.2967/jnumed.116.184333 28209905 PMC6944181

[8] Shin YW Lee ST Shin JW Moon J Lim JA Byun JI . VGKC-complex/LGI1-antibody encephalitis: clinical manifestations and response to immunotherapy. J Neuroimmunol. (2013) 265:75–81. doi: 10.1016/j.jneuroim.2013.10.005 24176648

[9] Kim TJ Lee ST Shin JW Moon J Lim JA Byun JI . Clinical manifestations and outcomes of the treatment of patients with GABAB encephalitis. J Neuroimmunol. (2014) 270:45–50. doi: 10.1016/j.jneuroim.2014.02.011 24662003

[10] Le WT Maleki F Romero FP Forghani R Kadoury S . Overview of machine learning: part 2: deep learning for medical image analysis. Neuroimaging Clin N Am. (2020) 30:417–31. doi: 10.1016/j.nic.2020.06.003 33038993

[11] Lv RJ Pan J Zhou G Wang Q Shao XQ Zhao XB . Semi-quantitative FDG-PET analysis increases the sensitivity compared with visual analysis in the diagnosis of autoimmune encephalitis. Front Neurol. (2019) 10:576. doi: 10.3389/fneur.2019.00576 31244751 PMC6563773

[12] Currie G Hawk KE Rohren E Vial A Klein R . Machine learning and deep learning in medical imaging: intelligent imaging. J Med Imaging Radiat Sci. (2019) 50:477–87. doi: 10.1016/j.jmir.2019.09.005 31601480

[13] Liu Z Li Z Qu J Zhang R Zhou X Li L . Radiomics of multiparametric MRI for pretreatment prediction of pathologic complete response to neoadjuvant chemotherapy in breast cancer: A multicenter study. Clin Cancer Res. (2019) 25:3538–47. doi: 10.1158/1078-0432.ccr-18-3190 30842125

[14] Liu Z Meng X Zhang H Li Z Liu J Sun K . Predicting distant metastasis and chemotherapy benefit in locally advanced rectal cancer. Nat Commun. (2020) 11:4308. doi: 10.1038/s41467-020-18162-9 32855399 PMC7452897

[15] Wei G Jiang P Tang Z Qu A Deng X Guo F . MRI radiomics in overall survival prediction of local advanced cervical cancer patients tread by adjuvant chemotherapy following concurrent chemoradiotherapy or concurrent chemoradiotherapy alone. Magn Reson Imaging. (2022) 91:81–90. doi: 10.1016/j.mri.2022.05.019 35636572

[16] Tang Z Zhang XY Liu Z Li XT Shi YJ Wang S . Quantitative analysis of diffusion weighted imaging to predict pathological good response to neoadjuvant chemoradiation for locally advanced rectal cancer. Radiother Oncol. (2019) 132:100–8. doi: 10.1016/j.radonc.2018.11.007 30825957

[17] Chen Y Qi Y Hu Y Qiu X Qiu T Li S . Integrated cerebellar radiomic-network model for predicting mild cognitive impairment in Alzheimer's disease. Alzheimers Dement. (2025) 21:e14361. doi: 10.1002/alz.14361 39535490 PMC11782160

[18] Le TK Comte V Darcourt J Razzouk-Cadet M Rollet AC Orlhac F . Performance and clinical impact of radiomics and 3D-CNN models for the diagnosis of neurodegenerative parkinsonian syndromes on 18 F-FDOPA PET. Clin Nucl Med. (2024) 49:924–30. doi: 10.1097/rlu.0000000000005392 39104036

[19] Jiang H Du Y Lu Z Wang B Zhao Y Wang R . Radiomics incorporating deep features for predicting Parkinson's disease in (123)I-Ioflupane SPECT. EJNMMI Phys. (2024) 11:60. doi: 10.1186/s40658-024-00651-1 38985382 PMC11236833

[20] Yin TT Cao MH Yu JC Shi TY Mao XH Wei XY . T1-weighted imaging-based hippocampal radiomics in the diagnosis of Alzheimer's disease. Acad Radiol. (2024) 31:5183–92. doi: 10.1016/j.acra.2024.06.012 38902110

[21] Zarei A Keshavarz A Jafari E Nemati R Farhadi A Gholamrezanezhad A . Automated classification of Alzheimer's disease, mild cognitive impairment, and cognitively normal patients using 3D convolutional neural network and radiomic features from T1-weighted brain MRI: A comparative study on detection accuracy. Clin Imaging. (2024) 115:110301. doi: 10.1016/j.clinimag.2024.110301 39303405

[22] Yang M Meng S Wu F Shi F Xia Y Feng J . Automatic detection of mild cognitive impairment based on deep learning and radiomics of MR imaging. Front Med (Lausanne). (2024) 11:1305565. doi: 10.3389/fmed.2024.1305565 38283620 PMC10811129

[23] Jang Y Lee ST Bae JY Kim TJ Jun JS Moon J . LGI1 expression and human brain asymmetry: insights from patients with LGI1-antibody encephalitis. J Neuroinflamm. (2018) 15:279. doi: 10.1186/s12974-018-1314-2 30253786 PMC6156957

[24] Gernert JA Sanzo L Zimmermann H Thaler FS Vogler L Gnorich JS . 18F]fluorodeprenyl-D2 PET can detect and monitor astrogliosis in anti-LGI1-IgG autoimmune encephalitis. Eur J Nucl Med Mol Imaging. (2026) 53:2054–68. doi: 10.1007/s00259-025-07531-5 41003762 PMC12860853

[25] Rorden C Bonilha L Fridriksson J Bender B Karnath HO . Age-specific CT and MRI templates for spatial normalization. Neuroimage. (2012) 61:957–65. doi: 10.1016/j.neuroimage.2012.03.020 22440645 PMC3376197

[26] Pagani M Oberg J De Carli F Calvo A Moglia C Canosa A . Metabolic spatial connectivity in amyotrophic lateral sclerosis as revealed by independent component analysis. Hum Brain Mapp. (2016) 37:942–53. doi: 10.1002/hbm.23078 26703938 PMC6867238

[27] Mao X Zhang D Ying D Yu J Ge Y Jia Z . Integration of glymphatic system function and hippocampal radiomics for diagnosis and conversion prediction of Alzheimer's disease. BMC Med Imaging. (2026) 26:318. doi: 10.1186/s12880-026-02390-4 42098640 PMC13321504

[28] Feng F Wang P Zhao K Zhou B Yao H Meng Q . Radiomic features of hippocampal subregions in Alzheimer's disease and amnestic mild cognitive impairment. Front Aging Neurosci. (2018) 10:290. doi: 10.3389/fnagi.2018.00290 30319396 PMC6167420

[29] Shen Y Wang RC Wang JX Li Y Xu YH Li YF . Progressive modeling of multiscale hippocampal subfield structural representation improves the prediction performance of mild cognitive impairment in type 2 diabetes. Eur Arch Psychiatry Clin Neurosci. (2026). doi: 10.1007/s00406-026-02224-y 41917441

[30] Van Griethuysen JJ Fedorov A Parmar C Hosny A Aucoin N Narayan V . Computational radiomics system to decode the radiographic phenotype. Cancer Res. (2017) 77:e104–7. doi: 10.1158/0008-5472.can-17-0339 29092951 PMC5672828

[31] Firat Z Er F Noyan H Ekinci G Ucok A Ulug AM . Discriminant analysis using MRI asymmetry indices and cognitive scores of women with temporal lobe epilepsy or schizophrenia. Neuroradiology. (2024) 66:1083–92. doi: 10.1007/s00234-024-03317-y 38416211

[32] Thomas I Westin J Alam M Bergquist F Nyholm D Senek M . A treatment-response index from wearable sensors for quantifying Parkinson's disease motor states. IEEE J BioMed Health Inform. (2018) 22:1341–9. doi: 10.1109/jbhi.2017.2777926 29989996

[33] Zhang F Wang Y Zhang X Alzheimer's Disease Neuroimaging I . Deep learning-based hippocampus asymmetry assessment for Alzheimer's disease diagnosis. Med Phys. (2025) 52:e17831. doi: 10.1002/mp.17831 40241310

[34] Herzog NJ Magoulas GD . Brain asymmetry detection and machine learning classification for diagnosis of early dementia. Sensors (Basel). (2021) 21:778. doi: 10.3390/s21030778 33498908 PMC7865614

[35] Abunada M Nierobisch N Ludovichetti R Simmen C Terziev R Togni C . Autoimmune encephalitis: Early and late findings on serial MR imaging and correlation to treatment timepoints. Eur J Radiol Open. (2024) 12:100552. doi: 10.1016/j.ejro.2024.100552 38327544 PMC10847996

[36] Qiao S Wu HK Liu LL Wang ML Zhang RR Han T . Clinical features and long-term outcomes of anti-leucine-rich glioma-inactivated 1 encephalitis: A multi-center study. Neuropsychiatr Dis Treat. (2021) 17:203–12. doi: 10.2147/ndt.s292343 33531809 PMC7846830

[37] Teng Y Li T Yang Z Su M Ni J Wei M . Clinical features and therapeutic effects of anti-leucine-rich glioma inactivated 1 encephalitis: A systematic review. Front Neurol. (2021) 12:791014. doi: 10.3389/fneur.2021.791014 35095736 PMC8791026

[38] Dubey D Pittock SJ Kelly CR McKeon A Lopez-Chiriboga AS Lennon VA . Autoimmune encephalitis epidemiology and a comparison to infectious encephalitis. Ann Neurol. (2018) 83:166–77. doi: 10.1002/ana.25131 29293273 PMC6011827

[39] Lu C Lin Y Wu M Chen J Meng H Chen S . Brain atrophy in autoimmune encephalitis: epidemiology, pathophysiology, clinical manifestations, treatment, and prognosis-an update. J Neurol. (2025) 273:32. doi: 10.1007/s00415-025-13571-6 41417104

