# Supplementary material for: Hippocampal texture asymmetry features on 18F-FDG PET differentiating anti-LGI1 and anti-GABABR encephalitis
Source: Front Immunol. 2026 Aug 25;17:1878467. doi: 10.3389/fimmu.2026.1878467 (PMC13550173; doi:10.3389/fimmu.2026.1878467)
Supplement: Supplementary file 1 [file Supplementaryfile1.docx]

Supplementary Material

# Supplementary Method 1: Sensitivity analysis across different feature selection and SMOTE sequences

To evaluate whether performing feature selection (FS) prior to SMOTE affects model performance and generalization on minority classes, this study compared three experimental scenarios:

1. Scenario (a) Baseline (No SMOTE): Model trained without any oversampling.
2. Scenario (b) SMOTE-FS: Applying SMOTE oversampling first across all raw extracted features, followed by feature selection.
3. Scenario (c) FS-SMOTE: Performing feature selection (variance filtering, univariate test, Pearson correlation, and LASSO) first on raw features, followed by SMOTE oversampling within the selected feature subset.

# Supplementary Result:

The evaluation metrics (AUC, Accuracy, Sensitivity, Specificity) across the three pipelines is summarized in **Supplementary Table 1**. Under Scenario (a), the baseline model achieved an AUC of 0.841 (0.625-1.000) and an accuracy of 73.68%. Under Scenario (b), the model achieved an AUC of 0.830 (0.602-1.000) and an accuracy of 78.95%. Under Scenario (c), the model achieved an AUC of 0.898 (0.743-1.000) and an accuracy of 84.21%. In addition, FS-SMOTE achieved the highest Sensitivity (81.82%), markedly outperforming both No SMOTE (63.64%) and SMOTE-FS (72.73%), while maintaining identical high Specificity (87.50%) across all three pipelines. These results indicated that FS-SMOTE substantially enhances, rather than underestimates, in detecting minority class cases.

**Supplementary Table 1**. Sensitivity analysis comparing model performance across different feature selection and SMOTE sequences on the testing cohort

| Scenario | AUC (95% CI) | Accuracy | Sensitivity | Specificity |
| --- | --- | --- | --- | --- |
| No SMOTE | 0.841 (0.625-1.000) | 73.68% | 63.64% | 87.50% |
| SMOTE-FS | 0.830 (0.602-1.000) | 78.95% | 72.73% | 87.50% |
| FS-SMOTE | 0.898 (0.743-1.000) | 84.21% | 81.82% | 87.50% |
